# MMP-9 Concentration in Peritoneal Fluid Is a Valuable Biomarker Associated with Endotoxemia in Equine Colic

**DOI:** 10.1155/2021/9501478

**Published:** 2021-01-05

**Authors:** Ann Kristin Barton, Ina-Gabriele Richter, Tanja Ahrens, Roswitha Merle, Abdollah Alalwani, Svenja Lilge, Katrin Purschke, Dirk Barnewitz, Heidrun Gehlen

**Affiliations:** ^1^Equine Clinic, Freie Universitaet Berlin, Berlin, Germany; ^2^Research Centre of Medical Technology and Biotechnology, Bad Langensalza, Germany; ^3^Institute for Veterinary Epidemiology, Freie Universitaet Berlin, Berlin, Germany

## Abstract

The purpose of the study was to compare the results of sepsis scoring (clinical examination and clinical pathology) to the concentrations of matrix-metalloproteinases (MMPs) -2, -8, and -9; tissue-inhibitor of metalloproteinases (TIMPs) -1 and -2; and inflammatory chemokines interleukin (IL) 1*β* and tumor-necrosis-factor-alpha (TNF-*α*) in plasma and peritoneal fluid of equine colic patients. A modified sepsis scoring including general condition, heart and respiratory rate, rectal temperature, mucous membranes, white blood cell count (WBC), and ionized calcium was applied in 47 horses presented with clinical signs of colic. Using this scoring system, horses were classified as negative (*n* = 32, ≤6/19 points), questionable (*n* = 9, 7-9/19 points), or positive (*n* = 6, ≥10/19 points) for sepsis. MMPs, TIMPs, IL-1*β*, and TNF-*α* concentrations were evaluated in plasma and peritoneal fluid using species-specific sandwich ELISA kits. In a linear discriminant analysis, all parameters of sepsis scoring apart from calcium separated well between sepsis severity groups (*P* < 0.05). MMP-9 was the only biomarker of high diagnostic value, while all others remained insignificant. A significant influence of overall sepsis scoring on MMP-9 was found for peritoneal fluid (*P* = 0.005) with a regression coefficient of 0.092, while no association was found for plasma (*P* = 0.085). Using a MMP-9 concentration of >113 ng/ml in the peritoneal fluid was found to be the ideal cutoff to identify positive sepsis scoring (≥10/19 points; sensitivity of 83.3% and specificity of 82.9%). In conclusion, MMP-9 was found to be a biomarker of high diagnostic value for sepsis and endotoxemia in equine colic. The evaluation of peritoneal fluid seems preferable in comparison to plasma. As abdominocentesis is commonly performed in the diagnostic work-up of equine colic, a pen-side assay would be useful and easy-to-perform diagnostic support in the decision for therapeutic intervention.

## 1. Introduction

The equine gastrointestinal flora is rich in gram-negative bacteria, which facilitate fermentation and digestive processes [1]. In health, only slight amounts of endotoxin, i.e., cell-wall components of these bacteria, reach the circulation due to the activity of specialized macrophages within the hepatic sinusoids, the so called Kupffer cells. In strangulating obstruction and severe gastrointestinal inflammation, for example, colitis, large amounts of endotoxin accumulate within the inflamed intestinal wall and reach the liver. When the phagocytocic capacity of Kupffer cells is overwhelmed, endotoxemia and possibly sepsis may develop [2].

In consequence, proteolytic enzymes are produced and activated, in particular matrix metalloproteinases (MMPs). The inflammatory response is mainly driven by the release of cytokines from macrophages and monocytes at the site of injury, followed by neutrophils releasing cytokines themselves. These proinflammatory mediators especially interleukin-1 (IL-1), tumor necrosis factor alpha (TNF-*α*), and interleukin-6 (IL-6) stimulate the hepatic acute-phase protein synthesis, once released into the circulation [3]. LPS, TNF-*α*, interleukin-8, and granulocyte-colony-stimulating factor (G-CSF) stimulate MMP production by neutrophils in humans and other species [4]. Reactive oxygen species from neutrophilic granules regulate the activity of vascular MMPs, which are activated after contact with LPS in men and horses [5–7], and it explains increased concentrations in plasma. This is one of the key mechanisms of hypotonia in sepsis in mice [8].

In health, a dynamic equilibrium between elastinolytic, collagenolytic activity, and its inhibition is maintained for physiological balance. This balance is controlled by proteolytic degradation of extracellular matrix components by MMPs and inhibition of MMP activity by specific tissue inhibitors of matrix metalloproteinases (TIMPs) [9, 10]. Increased levels of different MMPs and TIMPs have been shown in multiorgan disease with MMP-9 showing particularly high diagnostic value. Gelatinolytic activity of MMP-2 and MMP-9 is increased in human patients suffering from gram-negative sepsis [11]. Plasma concentrations of MMP-9 increase up to 8 hours after LPS infusion in human subjects and baboons [11, 12]. The same was evident in different organs of mice after LPS infusion [5, 8, 13]. In horses, LPS administration significantly increased MMP-2 and MMP-9 activities in plasma, which could be reduced by several nonsteroidal anti-inflammatory drugs (NSAIDs), in particular pentoxifylline and oxytetracycline [14]. MMP-9 was also shown to be a diagnostic indicator of survival during sepsis [15]. An association of sepsis-related mortality disease with TIMP-1/MMP-9 ratios was found in several studies for adults and children [16–20]. Increased concentrations in peritoneal fluid are likely to be produced by peritoneal macrophages in consequence of strangulating obstruction and exudation of inflammatory mediators from the intestine as shown in rats [5, 8, 13]. MMP-9, TIMP-1, and MMP-9/TIMP ratio were good diagnostic or prognostic biomarkers of sepsis after major abdominal surgery and were linked to sepsis-associated organ dysfunction in rats and humans [21, 22]. This was confirmed for other organ systems and sepsis-associated diseases including acute kidney disease, acute respiratory failure, febrile neutropenia, and burn injuries [23–26]. TIMP-1 levels also correlated with sepsis-associated coagulopathy, both with the degree of disseminated intravascular coagulation (DIC) and disease severity [27].

In former studies, we could demonstrate significant increases in MMP-2, MMP-8, and MMP-9 as well as TIMP-1 and TIMP-2 concentrations in bronchoalveolar lavage fluid of horses suffering from mild-to-moderate and severe equine asthma, which normalized during successful therapy [28, 29]. As MMP-8/TIMP ratios demonstrating collagenolytic misbalance were of highest diagnostic value, we were interested in the role of MMP-8 in extracelluar remodeling within the abdomen after colic surgery. In equine colic, increased concentrations of another gelatinolytic MMP, namely, MMP-2, were found in serum [30]. Other authors found similar results of increased MMP-2 and MMP-9 levels in equine arthritis and synovitis [31–33] as well as laminitis [34–36]. The exact role of MMP-9 in acute phases of laminitis remains uncertain though, as it appears to be present largely in its inactive form [37]. ECM remodeling also plays a role in ovulation and late gestation with MMP misbalance possibly inhibiting pregnancy or leading to a high-risk pregnancy or fetal membrane retention [38–40]. MMP-1 and possibly also MMP-2 and MMP-9 are overexpressed in neoplastic tissue of equine sarcoids [41, 42] and corneal ulceration [43, 44].

Abdominocentesis, followed by macroscopic, laboratory, and cytological evaluation of peritoneal fluid, is commonly performed in the work-up of equine colic and other gastrointestinal diseases. Quantifications of white and red blood cells, protein, lactate, and glucose concentrations are helpful to differentiate between strangulating and nonstrangulating disorders, in particular of the small intestine [45]. Levels in peritoneal fluid correlate with plasma concentrations, but increase earlier and may be useful to predict the need for surgery and estimate prognosis, especially using repeated sampling over time. Blood and peritoneal fluid supernatant TNF and IL-6 activities and endotoxin concentration were significantly greater in horses with colic, compared with healthy horses. In horses with colic, the peritoneal fluid endotoxin concentration and TNF and IL-6 activities were significantly greater than those in blood [46]. In addition, several studies have been published focusing on the increases in lactate dehydrogenase, creatine kinase, and alkaline phosphatase activity in comparison with lactate concentrations [47–52]. Peritoneal fluid phosphate concentrations also had reasonable sensitivity and specificity to predict intestinal lesions requiring resection or euthanasia [53]. Another promising marker for endotoxemia and equine colic might also be procalcitonin in plasma and peritoneal fluid [54, 55].

In this study, we aimed to evaluate the diagnostic value of several MMPs (MMP-2, MMP-9, MMP-8) and TIMPs (TIMP-1 and TIMP-2) for equine colic, which is often associated with endotoxemia and sepsis in case of strangulating obstruction. In addition, important regulators of the hepatic acute-phase protein synthesis (IL-1*β* and TNF-*α*) were evaluated, as strangulating obstruction may lead to fibrosis formation in consequence of elastinolytic or collagenolytic misbalance. A sepsis scoring system for the evaluation of endotoxemia in equine colic patients has been published [56]. Our hypothesis was that there is an association between sepsis scoring, local and systemic MMP, TIMP, and cytokine concentrations in equine colic. As prior studies found higher concentrations of inflammatory markers in local samples compared to plasma [46, 55, 57], we evaluated concentration in peritoneal fluid in addition to plasma.

## 2. Materials and Methods

### 2.1. Sepsis Scoring

47 horses presented to the Equine Clinic of Freie Universitaet Berlin were included in the study. All horses showed clinical signs of colic with various severities. The sepsis scoring system published by Breuer and Schusser [56] was modified to achieve the feasibility of sepsis scoring in all hours of the day (emergency care). In this modified version, it included general condition, heart and respiratory rate, rectal temperature, mucous membranes, white blood cell count (WBC), and ionized calcium counting up to 19 points maximum (Table 1). Scoring results ≤ 6/19 points were classified as negative for sepsis, 7-9 points as questionable, and ≥10/19 points as positive for sepsis.

### 2.2. Ethical Statement

All parameters included in the scoring system were taken during the routine diagnostic workup of colic in our clinic, and all MMP, TIMP, and chemokine concentrations in plasma and peritoneal fluid were evaluated from samples taken during routine puncture of the jugular vein and abdominocentesis for clinical pathology at admission or before surgery. Therefore, sampling of horses affected by colic was not classified as animal experiments by the State Office of Health and Social Affairs Berlin (LaGeSo). Owners' consent to involve their horses in the study was obtained during the admission process at the clinic.

### 2.3. Sample Taking Process

Blood samples were taken by venipuncture of the jugular vein, and peritoneal fluid was taken by abdominocentesis during the diagnostic workup upon arrival at the clinic. While routine hematology was performed using EDTA as a preservative, the remaining blood was transferred to Na-citrate and heparin-containing tubes to allow quantification of parameters studied. Plasma and cell-free supernatant of peritoneal fluid were obtained after centrifugation of the samples (10 minutes at 3500 U/min in Hettich Rotofix 32 centrifuge) and stored at -80°C until assayed.

### 2.4. ELISA of MMP-2, MMP-8, MMP-9, TIMP-1, TIMP-2, IL-1*β*, and TNF-*α*

The ELISAs used in this study were sandwich enzyme immunoassays for quantification of MMP, TIMP, and chemokine concentrations. Standards and samples were set up in duplicates. The concentrations in plasma and peritoneal fluid were determined using species-specific ELISA kits (ELISA Kit for Equine Matrix Metalloproteinase 2 (MMP-2), Cloud Clone Corp., USA. ELISA Kit for Equine Matrix Metalloproteinase 8 (MMP-8), Genorise Scientific Inc., USA. Equine Matrix Metalloproteinase 9 (Gelatinase B, 92 kDa Gelatinase, 92 kDa Type IV Collagenase) (MMP9) FAST ELISA Kit, fzmb GmbH, Germany. ELISA Kit for Equine Tissue Inhibitors of Metalloproteinase 1 (TIMP-1), Cloud Clone Corp., USA. ELISA Kit for Equine Tissue Inhibitors of Metalloproteinase 2 (TIMP-2), Cloud Clone Corp., USA. ELISA Kit for Equine Interleukin 1 beta (IL-1ß), Cloud Clone Corp., USA. ELISA Kit for Equine Tumor Necrosis Factor Alpha (TNF-*α*), Cloud Clone Corp., USA). An overview of the limits of detection and detection ranges is given in Table 2. The absorbance was measured with an ELISA microplate reader (ELISA microplate reader, Tecan, Switzerland.) at 450 nm immediately. Calculation of the unknown sample concentration was made by an inverted standard curve using the Excel software program.

### 2.5. Statistical Analysis

Data were statistically analyzed and graphically presented using SPSS Statistics® (version 25). The data were tested for normal distribution using the Shapiro Wilk test and visual inspection. To achieve normal distribution of data, MMP-9 and TIMP concentrations in plasma and peritoneal fluid were logarithmized to the basis of 10.

The influence of the overall sepsis score as well as single parameters on MMP-2/-8/-9, TIMP-1/-2, and IL-1*β*/TNF-*α* concentrations in plasma and peritoneal fluid, respectively, was investigated using a general linear regression model adjusted for confounding factors (age, breed, body weight, and gender). A stepwise backward selection was used to eliminate variables that did not contribute significantly to the model. The level of significance was set at *P* < 0.05. Multivariable ANOVA models were also fitted to investigate the influence of the sepsis score, MMP, TIMP, and chemokine concentrations on the total protein (TP), hematocrit (HCT), lactate or fibrinogen (concerning plasma values), and on TP and lactate (concerning peritoneal fluid values), also adjusted for potential confounders age, breed, body weight, and gender. A ROC analysis was performed to calculate the cutoff values for MMP-9 concentrations with the highest sensitivity and specificity concerning the detection of overall sepsis scoring ≥ 10. The discriminatory power of the single factors that contributed to the sepsis scoring was investigated using linear discriminant analysis. With this method, it can be evaluated which of the factors are well suited to discriminate between the sepsis scoring groups. The test of equality of group means was used to identify variables that discriminate well between the groups (*P* value <0.05). The assessment of the functions was done using the eigenvalue, the percentage of variance that is due to the function, and the respective *P* values of Wilks' lambda. The standardized canonical discriminant function coefficients were used to calculate the mean discriminant coefficients explaining the impact of the variables on the sepsis scoring groups.

## 3. Results

### 3.1. Sepsis Scoring

47 horses of our hospital population presenting with clinical signs of colic between March 2017 and January 2018 participated in the study. These horses were of various breeds (26 Warmbloods, 5 TB/SBs, 6 Western, 10 ponies), gender (26 mares, 14 geldings, 7 stallions), age (14.3 ± 7.9 years), and weight (512 ± 135 kg). According to the results of the sepsis scoring system, 32 horses were classified as negative (≤6/19 points), 9 as questionable (7-9/19 points), and 6 as positive (≥10/19 points) for sepsis. Horses negative for sepsis had a mean overall sepsis score of 2.8 ± 1.8, the questionable group had a mean score of 7.6 ± 0.7, and the positive group of 11.3 ± 2.0, respectively. An overview of the single parameters of sepsis scoring in the positive, questionable, and negative sepsis group is given in Table 3. All 6 horses with positive sepsis score went to surgery (4/6) or were euthanized due to financial restraints (2/6). During surgery, ischemic lesions were found in 4/4. Out of these, 3/4 survived until discharge; one was euthanized due to severe postoperative ileus and a decision of the owner against relaparotomy.

The discriminant analysis showed that all parameters of sepsis scoring apart from ionized calcium (*P* = 0.403) discriminated well between sepsis severity groups (*P* = 0.001 or lower). Wilks' lambda recommended using two discriminant functions although the first function already accounted for 90.6% of the total variance. Discoloration of mucous membranes turned out to have the largest discriminatory power (mean discriminant function 7.55), followed by leukopenia and general condition (mean discriminant function 4.86 and 2.49, resp.).

The color of mucous membranes had the largest impact on the sepsis score. Including only the color of mucous membranes leads to misclassification of only four horses, i.e., 12.5%, compared to overall scoring. Other important variables were leukopenia and the general condition. The combination of these three variables classified all horses except one correctly (3.1% misclassification) compared to overall scoring. All six sepsis-positive horses were classified correctly. Unfortunately, the number of sepsis positive horses was too small to calculate reliable cutoff values for overall sepsis scoring concerning the need for surgery, resection, or survival.

### 3.2. ELISA of MMP-2/-8/-9

MMP-2 concentrations in the group negative for sepsis were 55.3 (42.3–72.1) ng/ml in plasma and 7.8 (0.0–23.90) ng/ml in peritoneal fluid (PF), respectively. In the questionable group, MMP-2 concentrations were 47.0 (31.6–52.1) ng/ml in plasma and 11.4 (1.3–24.0) ng/ml in PF. In sepsis-positive horses, concentrations were 69.4 (54.7–84.4) ng/ml in plasma and 37.7 (0.0–90.4) ng/ml in PF with no significant differences between sepsis groups (*P* > 0.05).

Very low concentrations were measured for MMP-8: 0.80 (0.00–1.99) ng/ml in plasma and 0.00 (0.00–0.30) ng/ml in PF in the group negative for sepsis, 1.60 (0.45–2.40) ng/ml in plasma and 0.29 (0.00–0.75) ng/ml in PF in the questionable group, and 1.79 (0.00–5.47) ng/ml in plasma and 0.05 (0.00–1.85) ng/ml in PF in horses tested positive for sepsis. Again, no differences were found between sepsis groups (*P* > 0.05).

In the group negative for sepsis, MMP-9 concentrations were 107.8 (75.8–134.3) ng/ml in plasma and 51.9 (18.9–74.8) ng/ml in PF, respectively. In the questionable group, MMP-9 levels were 86.0 (67.5–105.4) ng/ml in plasma and 26.0 (7.5–182.1) ng/ml in peritoneal fluid. A significant increase of both parameters was found in the group scoring positive for sepsis with 143.8 (113.8–672.0) ng/ml in plasma (*P* = 0.011) and 954.6 (119.4–8268.5) ng/ml in peritoneal fluid (*P* = 0.002). An overview of the results of MMP concentrations in plasma and peritoneal fluid in the positive, questionable, and negative sepsis group is given in Table 4.

### 3.3. ELISA of TIMP-1/-2

TIMP-1 concentrations in the group negative for sepsis were 472.4 (289.4–765.1) ng/ml in plasma and 223.7 (180.4–272.9) ng/ml in PF, respectively. In the questionable group, TIMP-1 concentrations were 723.5 (357.7–1295.2) ng/ml in plasma and 204.7 (188.1–389.2) ng/ml in PF. In sepsis-positive horses, concentrations were 907.2 (755.6–1114.3) ng/ml in plasma and 512.9 (448.2–1024.4) ng/ml in PF with no significant differences between sepsis groups.

In the group negative for sepsis, TIMP-2 concentrations were 86.5 (63.9–265.0) ng/ml in plasma and 0.0 (0.0–30.1) ng/ml in peritoneal fluid, respectively. In the questionable group, TIMP-2 levels were 98.3 (0.0–139.2) ng/ml in plasma and 0.0 (0.0–0.0) in peritoneal fluid. In sepsis-positive horses, concentrations were 133.7 (61.2–360.2) ng/ml in plasma and 25.2 (0.0–285.3) ng/ml in PF with no significant differences between sepsis groups.

An overview of the results of TIMP concentrations in plasma and peritoneal fluid in the positive, questionable, and negative sepsis group is given in Table 5.

### 3.4. ELISA of IL-1*β* and TNF-*α*

Very low concentrations were found for IL-1*β* and TNF-*α*. Concentrations of IL-1*β* were 2.26 (2.20–2.50) pg/ml in plasma and 2.40 (2.23–2.68) pg/ml in PF in the group negative for sepsis, 2.20 (1.60–2.20) pg/ml in plasma and 2.45 (1.68–2.57) pg/ml in PF in the questionable group, and 2.29 (2.20–2.34) pg/ml in plasma and 2.59 (2.42–2.95) pg/ml in PF in horses tested positive for sepsis. No differences were found between sepsis groups.

Concentrations of TNF-*α* were 7.9 (7.9–13.0) pg/ml in plasma and 7.9 (7.9–8.1) pg/ml in PF in the group negative for sepsis, 7.9 (6.7–7.9) pg/ml in plasma and 7.9 (6.7–10.5) pg/ml in PF in the questionable group, and 8.9 (7.9–32.3) pg/ml in plasma and 22.1 (7.9–34.2) pg/ml in PF in horses tested positive for sepsis. Again, no differences were found between the sepsis groups.

An overview of the results of IL-1*β* and TNF-*α* concentrations in plasma and peritoneal fluid in the positive, questionable, and negative sepsis group is given in Table 6.

### 3.5. Statistical Analysis

A significant influence of overall sepsis scoring on log MMP-9 was found for peritoneal fluid (*P* = 0.005) with a regression coefficient of 0.092, while no significant association was found for plasma (*P* = 0.085). Log TIMP-1 in the peritoneal liquid was significantly influenced by overall scoring (*P* = 0.018).

In blood, TIMP-1 was associated with HCT (regression coefficient 9.447, *P* = 0.024). Lactate in blood was influenced by MMP-9 (*P* = 0.012, regression coefficient 0.836). For TP, no statistically significant factors could be identified.

For MMP-2, MMP-8, TIMP-2, IL-1*β*, and TNF-*α*, no significant differences were found between the sepsis groups, and no correlations were found to the 8 parameters of overall sepsis scoring classifying horses as septic (≥10/19 points) or nonseptic (≤9/19 points) or the 3 subgroups (negative, questionable, positive). There was a positive association of MMP-8 and lactate in plasma, one rising with the other, but this trend remained insignificant (*P* = 0.088). There was also a trend for IL-1*β* and lactate in PF (*P* = 0.053). A significant positive association was found between IL-1*β* and TP in PF (*P* = 0.023).

ROC analyses were used to find the optimal cutoff of MMP-9 as well as TIMP-1 concentrations to identify animals with positive sepsis scoring (≥10/19 points). A cutoff is regarded as optimal when it has high sensitivity and high specificity. This can be evaluated by calculating sensitivities and specificities for a range of MMP-9 concentrations and assess the results visually by a ROC graph. Regarding MMP-9 concentrations in peritoneal fluid, 113 ng/ml turned out to be optimal, since the sensitivity was 83.3% and the specificity was 82.9%. For MMP-9 in plasma, the best concentration was 109 ng/ml with a sensitivity of 100%, but a specificity of only 60%. For TIMP in peritoneal fluid, the optimal cutoff value was 374 ng/ml, which showed a sensitivity of 83.3% and a specificity of 91.4%. For TIMP values in blood, 541 ng/ml turned out to be the optimal cutoff value with a sensitivity of 100% and a specificity of 57.1%.

## 4. Discussion

In this study, only MMP-9 was found to be a biomarker of high diagnostic value for sepsis and endotoxemia in equine colic. The evaluation of peritoneal fluid seems preferable in comparison with plasma.

Apart from the overrepresentation of stallions, the study population can be regarded as representative of our hospital population of equine colic. The sepsis scoring was modified from Breuer and Schusser [56] to increase its feasibility during all hours of the day (emergency care). Blood cultures for example are currently not a part of our routine diagnostic workup of colic emergencies arriving at the clinic; therefore, we decided to delete the parameter “infection” and reduced the points leading to classification as negative, questionable, or positive for sepsis accordingly. The results of the discriminant analysis showed that all parameters apart from ionized calcium contributed to the discrimination between sepsis groups alone; therefore, we think that modification of the score is defensible. The results of the discriminant analyses showed that it would be possible to reduce the number of variables that account for the score even further. Inclusion of only the color of mucous membranes, general condition, and leukopenia would increase the risk of misclassification only slightly. Nevertheless, we decided to go on with the overall scoring to have the best statistical power when evaluating the regression of MMP-9 concentrations in plasma or peritoneal fluid and sepsis scoring.

Our hypothesis that there is a regression of local and systemic MMP-9 concentrations and endotoxemia in equine colic was confirmed. The regression coefficient for MMP-9 in plasma was weak though and the regression remained insignificant, which is in unison with prior studies in other species and organ systems, where higher concentrations were found in local samples compared to plasma as well [28–33, 46–53]. Therefore, this was not unexpected and the reason why we evaluated MMP-9 concentration in peritoneal plasma in addition to plasma. Apart from peritoneal macrophages, MMPs are likely to be produced by neutrophils in septic or even nonseptic peritonitis in consequence of strangulating obstruction and following the upregulation of inflammatory mediators like TNF-*α*, interleukin-8, and G-CSF [4].

Interestingly, the mean MMP-9 concentration in the group questionable for sepsis was lower than in the negative group, which was visible in plasma and peritoneal fluid. This may be simply caused by the much smaller number of horses in the questionable versus the negative group; another explanation may be that the synchronous activation of the elastinolytic system may have reduced inflammatory mediators favoring MMP production in the early phase of inflammation. At least in plasma, this is supported by TIMP level increases in the questionable group, in particular TIMP-1.

During the calculation of optimal cutoff values for MMP-9 concentration in plasma and peritoneal fluid, it became obvious that these would be quite low in comparison with mean values in horses scoring negative or questionable for sepsis. The cutoff of 113 ng/ml in peritoneal fluid had reasonable sensitivity and specificity in comparison with that of plasma. As there was no regression between sepsis scoring and MMP-9 concentrations in plasma, quantification of MMP-9 in plasma cannot be recommended for clinical routine to aid the diagnosis of strangulating obstruction or to help with the decision for surgery, antibiotics, or euthanasia. On the other hand, a stable side or quick assay for MMP-9 evaluation from peritoneal fluid would be very interesting, as abdominocentesis is commonly performed in horses presenting with colic and unclear findings in clinical examination, transrectal palpation, or abdominal ultrasonography. Other parameters from peritoneal fluid, i.e., lactate, are very established in clinical routine regarding the decision for surgery and the assumption of prognosis, but increases in MMP-9 indicating gelatinolytic disbalance may be an early prognostic indicator for fibrosis formation, which should be evaluated in longitudinal studies. As MMPs can be regarded as markers associated with coagulation and fibrinolysis, they could correlate with strangulating obstruction, need for surgery, and survival in equine colic, where a misbalance of coagulation and fibrinolysis was demonstrated in several studies [58–61].

In humans, expression of MMP-9 during sepsis and endotoxemia is known for a longer time [62]. LPS induces transcription of the MMP-9 gene, and several groups have investigated the levels of MMP-9 in relation to the severity of sepsis in humans. Elevated levels of MMP-9 were observed in critically ill patients [63], and a relation of MMP-9 levels to the severity of sepsis was shown [3]. In a clinical study of patients with septic shock, MMP-9 levels in nonsurvivors were higher than those in survivors and healthy controls [15]. The hub genes for MMP-9 were significantly related to the prognosis of sepsis patients [64]. Latest studies in human medicine suggest not only a coincidence but a promoting role of MMPs in inflammation as shown in invasion and migration of tumor cells [65].

The other parameters studied (MMP-2/-8, TIMP-1/-2, IL-1*β*, and TNF-*α*) remained quite unrewarding, unfortunately. Therefore, conclusions on an elastinolytic or collagenolytic misbalance cannot be realistically drawn at this point of time. It was very difficult to find suitable ELISA kits for equine samples. The manufacturers were asked for the immunization antigens and validation data. The manufacturers explained the use of recombinant horse TIMP and MMP as immunogenic and assured the minor validation with horse samples. All ELISA kits were sold as validated equine-specific tests as shown in Table 2. Nevertheless, all tests apart from the MMP-9 kit had problems concerning missing-specific native equine enzymes and proteins (mostly recombinant proteins were used as immunization antigens and ELISA standards). We relied on these tests after the manufacturer's recommendation. Furthermore, we did not find major problems in prior studies using equine BALF and kits from the same manufacturer and therefore did not include zymography again. The possibility remains that the failure to show any significant differences for the other assessments besides MMP-9 may be a matter of sensitivity. Unfortunately, due to the lack of fully validated assays, which are accredited for a diagnostic approach in veterinary medicine, we cannot see a loophole out of this dilemma at this point of time. Worth mentioning apart from this, the samples were taken at a very early time point prior to colic surgery. It is surprising though that cytokines like IL-1*β* and TNF-*α* did not show any differences correlating to the severity of clinical signs or laboratory findings. It may be more informative to study samples from the postoperative period to gain information on extracellular remodeling within the abdominal cavity favoring adhesion and fibrosis formation.

In conclusion, MMP-9 concentrations were increased in plasma and peritoneal fluid of horses presenting with colic and scoring positive for sepsis. Based on the results of this primary project and so far only 6 horses classified as sepsis-positive, MMP-9 may be useful to evaluate the severity of endotoxemia and may have prognostic value for equine colic. In further planned clinical studies, the overall specificity and sensitivity of MMP-9 for sepsis diagnosis should be determined by receiver operating characteristic (ROC) for a bigger number of horses with clearly defined sepsis (sepsis score with proof of infection by blood culture or PCR) of further origin than colic, in particular inflammatory gastrointestinal disease. Here, we aim to investigate the role of MMP-9 for (mortality-) prognosis or indication for surgery in case of colic and also the possible use of MMP-9 for antibiotic stewardship during postoperative care and the management of complications like peritonitis or postoperative ileus.

## Figures and Tables

**Table 1:** Sepsis scoring modified from Breuer and Schusser [56]: classification in negative (≤6/19 points), questionable (7-9/19 points), or positive (≥10/19 points) for sepsis.

Parameter	0	1	2	3
General condition	Bright, alert	Mildly reduced	Moderately reduced	Severely reduced
Heart rate/min	28-40	41-60	61-80	>80
Respiratory rate/min	10-14	15-25	26-45	>45
Temperature (°C)	37.5-38.0	37.0-37.4 or 38.1-38.5	36.5-36.9 or 38.6-39.0	<36.5 or >39.0
Mucous membranes	Pinkish	Mildly reddish	Moderately reddish	Highly reddish, toxic line
Ionized calcium (mmol/l)	1.5–1.8	<1.5		
WBC (G/l)	4.9–12.6	4.0–4.8 or 12.7–15.0	3.0–3.9 or 15.1–20.0	<3.0 or >20.0

**Table 2:** Species, limits of detection, and detection ranges of ELISA kits used for quantification of matrix-metalloproteinase (MMP), tissue inhibitors of metalloproteinase (TIMP), interleukin (IL)-1*β*, and tumor necrosis factor-alpha (TNF-*α*) concentration in equine plasma and peritoneal fluid. Details on manufacturers are given in the footnotes.

Parameter	Species	Limit of detection	Detection range
MMP-2^a^	Horse	0.56 ng/ml	1.56–100 ng/ml
MMP-8^b^	Horse	18 pg/ml	93–6000 pg/ml
MMP-9^c^	Horse	0.25 ng/ml	0.5–24 ng/ml
TIMP-1^d^	Horse	0.241 ng/ml	0.625–40 ng/ml
TIMP-2^e^	Horse	3.1 ng/ml	7.8–500 ng/ml
IL-1*β*^f^	Horse	5.7 pg/ml	15.6–1000 pg/ml
TNF-*α*^g^	Horse	2.9 pg/ml	7.8–500 pg/ml

**Table 3:** Results of sepsis scoring in the groups negative (*n* = 32, ≤6/19 points), questionable (*n* = 8, 7-9/19 points), or positive (*n* = 6, ≥10/19 points) for sepsis; median (lower quantile–upper quantile). Significant differences in comparison with the sepsis negative group are marked as ^∗^.

Parameter	Negative	Questionable	Positive
General condition	0 (0–1)	2 (1–3)	3 (2–3)^∗^
Heart rate	1 (0–1)	1 (1–2)	2 (1–2)
Respiratory rate	1 (0–1)	2 (1–2)	1 (1–2)
Temperature	1 (0–1)	0 (0–1)	2 (2–3)
Mucous membranes	0 (0–0)	1 (1–1)	2 (2–3)^∗^
Ionized calcium	0 (0–0)	0 (0–0)	0 (0–1)
WBC	0 (0–0)	2 (0–3)	0 (0–3)
Overall score	3 (2–4)	8 (7–8)	10 (10–12)^∗^

**Table 4:** MMP-2, MMP-8, and MMP-9 concentrations in plasma and peritoneal fluid in the groups negative (*n* = 32, ≤6/19 points), questionable (*n* = 8, 7-9/19 points), or positive (*n* = 6, ≥10/19 points) for sepsis; median (lower quantile–upper quantile). Significant differences in comparison with the sepsis negative group are marked as ^∗^.

Parameter	Negative	Questionable	Positive
MMP-2 (ng/ml) in plasma	55.3 (42.3–72.1)	47.0 (31.6–52.1)	69.4 (54.7–84.4)
MMP-2 (ng/ml) in peritoneal fluid	7.8 (0.0–23.90)	11.4 (1.3–24.0)	37.7 (0.0–90.4)
MMP-8 (ng/ml) in plasma	0.80 (0.00–1.99)	1.60 (0.45–2.40)	1.79 (0.00–5.47)
MMP-8 (ng/ml) in peritoneal fluid	0.00 (0.00–0.30)	0.29 (0.00–0.75)	0.05 (0.00–1.85)
MMP-9 (ng/ml) in plasma	107.8 (75.8–134.3)	86.0 (67.5–105.4)	143.8 (113.8–672.0)^∗^
MMP-9 (ng/ml) in peritoneal fluid	51.9 (18.9–74.8)	26.0 (7.5–182.1)	954.6 (119.4–8268.5)^∗^

**Table 5:** TIMP-1 and TIMP-2 concentrations in plasma and peritoneal fluid in the groups negative (*n* = 32, ≤6/19 points), questionable (*n* = 8, 7-9/19 points), or positive (*n* = 6, ≥10/19 points) for sepsis; median (lower quantile–upper quantile). No significant differences.

Parameter	Negative	Questionable	Positive
TIMP-1 (ng/ml) in plasma	472.4 (289.4–765.1)	723.5 (357.7–1295.2)	907.2 (755.6–1114.3)
TIMP-1 (ng/ml) in peritoneal fluid	223.7 (180.4–272.9)	204.7 (188.1–389.2)	512.9 (448.2–1024.4)
TIMP-2 (ng/ml) in plasma	86.5 (63.9–265.0)	98.3 (0.0–139.2)	133.7 (61.2–360.2)
TIMP-2 (ng/ml) in peritoneal fluid	0.0 (0.0–30.1)	0.0 (0.0–0.0)	25.2 (0.0–285.3)

**Table 6:** IL-1*β* and TNF-*α* concentrations in plasma and peritoneal fluid in the groups negative (*n* = 32, ≤6/19 points), questionable (*n* = 8, 7-9/19 points), or positive (*n* = 6, ≥10/19 points) for sepsis. Median (lower quantile–upper quantile). No significant differences.

Parameter	Negative	Questionable	Positive
IL-1*β* (pg/ml) in plasma	2.26 (2.20–2.50)	2.20 (1.60–2.20)	2.29 (2.20–2.34)
IL-1*β* (pg/ml) in peritoneal fluid	2.40 (2.23–2.68)	2.45 (1.68–2.57)	2.59 (2.42–2.95)
TNF-*α* (pg/ml) in plasma	7.9 (7.9–13.0)	7.9 (6.7-7.9)	8.9 (7.9–32.3)
TNF-*α* (pg/ml) in peritoneal fluid	7.9 (7.9–8.1)	7.9 (6.7–10.5)	22.1 (7.9–34.2)

## Data Availability

The data supporting the results of the study will be deposited on the data repository of Freie Universitaet Berlin (Refubium).
